# A Computational Framework to Evaluate Interactions of BPA and Its Analogs with Human Liver X Receptor-Beta for Health Risk Assessment

**DOI:** 10.1021/acs.chemrestox.5c00460

**Published:** 2026-02-16

**Authors:** Rajesh Kumar Pathak, Saurav Kumar, Vikas Kumar

**Affiliations:** † 16777 Pere Virgili Institute for Health Research (IISPV), Tarragona 43005, Spain; ‡ Department of Chemical Engineering, Rovira i Virgili University, Tarragona 43007, Spain; § German Federal Institute for Risk Assessment (BfR), Berlin 10589, Germany

## Abstract

Bisphenols are widely used in industrial applications to produce plastics and other consumer products. Among them, bisphenol A (BPA) is the most extensively studied due to its well-documented endocrine-disrupting effects and its association with various health conditions, including metabolic disorders and liver disease. Due to its known toxicity, BPA use has been restricted in many countries, leading to the emergence of several structural analogs. Recent studies have shown that BPA can interfere with normal liver metabolism by interacting with Liver X Receptor-beta (LXRβ). Although some BPA analogs have also been reported to cause toxicity, their exact effects on LXRβ remain unclear. In this study, we investigated the interaction between BPA analogs and LXRβ using molecular docking. BPA and the known LXRβ ligand G58 were used as reference compounds. The top 10 BPA analogs were further evaluated for their pharmacokinetics and pharmacodynamics properties. Molecular dynamics simulations over 100 ns were performed to study the dynamic behavior of LXRβ in complex with these analogs. Binding free energies were then calculated using the MM-PBSA method. Our results showed that several BPA analogs exhibited predicted stronger binding activities to LXRβ than BPA. Although some analogs shared similar pharmacokinetic and pharmacodynamic profiles with BPA, their stronger interaction with LXRβ raises concerns about their potential hepatotoxicity. This study employs a robust *in silico* framework to predict that commonly used BPA alternatives may pose a greater potential hepatotoxic risk than the banned parent compound, highlighting the value of computational approaches in prioritizing chemicals for further experimental assessment.

## Graphical abstract

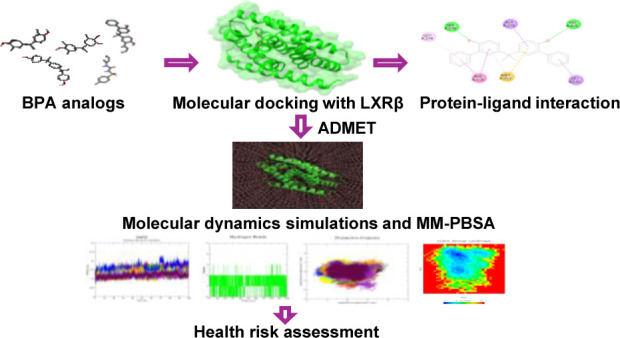

## 1 Introduction

Bisphenol A (BPA) is a well-established endocrine disruptor affecting human health due to persistent environmental exposure.^1^ Global restrictions on BPA have inadvertently driven the adoption of structurally similar analogs. This creates a public health paradox, since these substitutes may pose equal or greater metabolic risks than BPA itself.^2^ Over 200 BPA analogs have now been detected in human biomonitoring studies worldwide, with annual production exceeding 200,000 tons.^3–5^ However, a critical knowledge gap remains, it is unclear whether these “safer” alternatives are truly safe, which undermines regulatory intent.^2,6^ Recent evidence reveals that several BPA analogs accumulate in biological systems with potencies comparable to or exceeding BPA itself.^7–9^ Yet, regulatory frameworks globally lag behind scientific evidence. This lag allows most BPA analogs to enter commerce without a comprehensive safety evaluation of their interactions with critical metabolic targets. This regulatory-scientific disconnect demands immediate attention, particularly for understanding how these compounds interact with nuclear receptors governing liver metabolism, a system already under siege from the global epidemic of metabolic disorders.^7,10,11^ Being an important chemical for industrial use, BPA is utilized in industries for manufacturing of various daily used products such as plastic water bottles, plastic baby items, plastic food storage and packaging, thermal receipt paper, etc.^12,13^ Due to plastic packaging, BPA can leach into food. In addition, when waste materials degrade, it can be released into the environment, leading to contamination and ecological pollution.^14,15^ Humans are exposed to BPA through various routes such as oral intake, skin contact, and inhalation. Moreover, the production and use of BPA also contribute to the pollution of air and water.^1,16,17^ BPA has been detected at various concentrations in human blood, breast milk, and amniotic fluid.^15^ Several studies have also suggested a link between BPA exposure and reproductive disorders, metabolic diseases, and hormone-dependent cancers.^18,19^ After careful investigation of the toxic effects of BPA, its analogs became available for industrial applications and were considered as safe for human health to fulfill the demand of people. However, many studies have suggested that BPA analogs have similar toxic effects on the environment. Additionally, studies have reported that some BPA analogs are more dangerous than BPA.^15,20–23^

The liver is among the body’s largest and most metabolically active organs, sustaining life through protein synthesis, metabolic processing, endocrine control, and detoxification.^24^ It can carry out the biotransformation of many toxic chemicals. However, some harmful substances may still accumulate in the liver. These toxic substances, entering the body, may reach the liver through the blood, posing a threat to its function and health. The liver plays a crucial role in the metabolism of BPA. When BPA enters the human body, it is inactivated through a process called glucuronidation and is eventually excreted.^24–26^ Several studies have confirmed that BPA disrupts liver cell metabolism by inducing endoplasmic reticulum stress, increasing the production of reactive oxygen species, and reducing the β-oxidation of fatty acids.^24,27–29^ *In vitro* evidence indicates that BPA induces dose/time-dependent triglyceride accumulation in adipocytes and human liver cancer cells, driving cellular dysfunction and insulin resistance.^24,30^

As ligand-activated transcription factors, nuclear receptors modulate genes governing metabolism, growth, inflammation, and development.^24,31,32^ Liver X Receptor (LXR), a member of the nuclear receptor superfamily, plays a crucial role in cholesterol, glucose, and lipid metabolism, as well as in inflammation. Additionally, this receptor is implicated in liver diseases.^33,34^ Studies confirm that BPA interferes with liver fat processing and causes oxidative damage to mitochondria.^35^ At low doses, it upregulates LXR mRNA levels, which, in turn, affects liver lipid metabolism.^24,36^ In nonobese adults, BPA has also been shown to reduce blood glucose levels.^24,37^ LXR has two isoforms, Liver X Receptor-alpha (LXRα) and Liver X Receptor-beta (LXRβ).^33^ LXRβ is a critical molecular target; its disruption can cascade into metabolic dysfunction, liver steatosis, and systemic health impacts affecting millions globally.^34,38^ LXRβ’s unique dual role in regulating both cholesterol homeostasis and inflammatory responses establishes it as a key sentinel for metabolic disruption.^34,39–41^ Clarifying whether and how BPA analogs interact with LXRβ is thus urgent.^42^ Such insights are vital for preventing a new wave of chemical-induced metabolic disease.^24^

BPA dispute reveals an important divide in toxicology. Regulators tend to rely on consistent dose–response relationships when assessing risk, whereas many researchers place greater emphasis on biological relevance, particularly when considering hormone-related effects.^43^ Washington State’s Safer Products Program offers a logical alternative by prioritizing feasible substitutes over defining “safe” exposure levels (https://www.pugetsoundinstitute.org/bpa-toxicity-debate-approaches-regulatory-decisions-at-both-state-and-federal-levels/ accessed on 23/06/2025). Resolving contradictions requires harmonizing assay protocols, acknowledging nonmonotonic responses, and preventing regrettable substitutions through premarket toxicity screening.^7,44^

Previous studies demonstrated that many BPA analogs, such as Bisphenol S, Bisphenol F, Bisphenol AF, and Bisphenol B, can bind to endocrine and metabolic receptors with binding affinities comparable to or even higher than BPA.^23,42,45^ While most of these results are predictive, *in silico* findings help prioritize analogs for experimental investigation, reducing both cost and time.^46–48^ To further strengthen the biological relevance, the Adverse Outcome Pathway (AOP) framework was analyzed using AOPWIKI-EXPLORER. This approach illustrates how molecular initiating events, such as receptor binding by BPA analogs, can lead to key events and ultimately adverse outcomes like liver steatosis. The AOP perspective supports the mechanistic interpretation of our proposed objective and highlights the potential health risks associated with these analogs (Figure S1, Supporting Information).^49^

It is still not well understood how BPA affects LXR in a way that influences hepatic glucose and lipid metabolism.^24^ Understanding these interactions is critical for evidence-based chemical regulation and protecting public health from emerging metabolic threats. Efforts have been made in the present study to predict the interaction of BPA and its analogs (*n* = 22) with Liver X Receptor-beta (LXRβ) through molecular docking. Furthermore, the top 10 compounds were selected based on their predicted binding energy with LXRβ to perform ADMET prediction, molecular dynamics simulation, and Molecular Mechanics/Poisson–Boltzmann Surface Area (MM/PBSA) binding energy calculations to evaluate the interactions. These findings improve our understanding of the interactions between LXRβ and BPA analogs, which could support risk assessment efforts and help in the design of new therapeutics through structure-based drug discovery.

## 2 Materials and Methods

### 2.1 Structure Retrieval and Preparation

In the present study, 21 BPA analogs were selected based on their well-documented environmental prevalence and frequent use in “BPA-free” products, as reported in previous studies.^15^ The 3D structure of BPA and its analogs was retrieved from the PubChem database (https://pubchem.ncbi.nlm.nih.gov/) in structure-data file (SDF) format and OpenBabel (https://openbabel.org/) was used for minimizing and converting them to Protein Data Bank (PDB), partial charge (Q), and atom type (T) (PDBQT) file formats for molecular docking. The 3D structure of human LXRβ (PDB ID: 3L0E), crystallized with the G58 antagonist at a resolution of 2.30 Å, was retrieved from the Protein Data Bank (PDB) and visualized using UCSF Chimera (https://www.rcsb.org/).^50,51^

### 2.2 Protein Preparation and Receptor Grid Generation

The LXRβ was prepared in UCSF Chimera^50^ by removing nonstandard residues and the cocrystallized ligand G58. After adding partial charges and polar hydrogens in AutoDock Tools and converting the PDB file to PDBQT format, a grid box was generated around G58’s binding pocket.^52,53^ We established a 40 Å grid box (x, y, z) centered at coordinates 12.267, −36.043, and −4.402 Å for docking simulations. To verify our parameters, we used AutoDock Vina v1.2.5 to redock the cocrystallized ligand into its binding site.^54,55^ The root-mean-square deviation (RMSD) between the original ligand G58 and its redocked conformation was then calculated in PyMOL (https://pymol.org/).

### 2.3 Molecular Docking

The interaction study of BPA analogs with LXRβ was conducted through molecular docking using AutoDock Vina v1.2.5.^54,55^ The cocrystallized G58 and BPA were used as reference compounds. The protein–ligand complex was prepared and analyzed by Chimera software.^50^ Furthermore, 2D and 3D interaction plots of BPA and its analogs with LXRβ were generated by Discovery Studio Visualizer 2025 to decode the key amino acid residues of LXRβ involved in the interaction (https://discover.3ds.com/discovery-studio-visualizer-download). Since true negative control compounds are difficult to define—a known limitation for specificity assessment—we evaluated relative binding potential by comparison with established positive controls (G58 and BPA).^24,42^

### 2.4 Pharmacokinetics Analysis and Toxicity Risk Prediction

The SMILES notations of the top 10 prioritized BPA analogs, along with BPA itself and G58, were obtained from PubChem and analyzed using pkCSM (https://biosig-lab-uq-edu-au.sabidi.urv.cat/pkcsm/) to evaluate their pharmacokinetic profiles and potential toxicity. While pkCSM provides valuable preliminary pharmacokinetic and toxicity predictions, its dependence on *in silico* models without experimental validation represents a recognized limitation.^56,57^ As computational approaches cannot fully capture biological complexity, this may affect prediction accuracy for certain parameters.^57^ Our computational analysis evaluated key ADMET parameters such as absorption, distribution, metabolism, excretion, and toxicity. Specific endpoints included water solubility, blood–brain barrier permeability, interactions with key CYP enzymes, total clearance, and standard toxicity endpoints like the AMES test.^56,58^

### 2.5 Molecular Dynamics Simulation

Molecular dynamics simulations were performed on the top 10 BPA analogs-LXRβ complexes, along with the apo form of LXRβ, the LXRβ–BPA complex, and the LXRβ–G58 complex. Simulations were carried out using GROMACS v2022.4 with GPU acceleration, applying the CHARMM27 all-atom force field.^60,59^ Ligand topologies were generated via SwissParam.^61^ Each protein–ligand system was placed in a dodecahedral water box and solvated using the TIP3P water model. Counterions were incorporated to balance the system’s overall charge, followed by energy minimization using the steepest descent algorithm. The systems were then equilibrated under NVT and NPT ensembles to stabilize temperature, pressure, and volume.^59,60,62^ All simulations were run for 100 ns. To evaluate structural behavior, we employed root-mean-square deviation (RMSD) for assessing overall protein conformational stability, root-mean-square fluctuation (RMSF) for quantifying residue flexibility, and radius of gyration (Rg) for monitoring molecular compactness. Solvent-accessible surface area (SASA) analysis tracked folding and conformational changes, while hydrogen bond (HB) analysis characterized protein–ligand interactions. Large-scale motions were explored via principal component analysis (PCA) using GROMACS utilities (gmx rms, rmsf, gyrate, sasa, hbond, covar, anaeig), with trajectories visualized as 2D plots in Grace (https://plasma-gate.weizmann.ac.il/Grace/). Finally, free energy landscape (FEL) calculations using gmx sham identified the most stable conformational states of LXRβ and its complexes.

### 2.6 Binding Energy Calculation

Binding free energy calculations were performed to assess the affinity of BPA analogs for LXRβ using the MM-PBSA method.^63^ MM-PBSA is a widely employed computational approach for ranking ligand–receptor interactions; however, it comes with well-known limitations. In particular, the use of an implicit solvent model and the approximate treatment of entropic contributions can lead to the systematic overestimation of absolute binding free energies.^64–66^ Molecular dynamics simulation trajectory frames from the last 10 ns were used to compute the binding free energies of the selected protein–ligand complexes using the gmx_MMPBSA tool.^67^ Various energy components were calculated, including van der Waals energy, electrostatic energy, Poisson–Boltzmann energy, polar solvation energy, gas-phase free energy, solvation free energy, and the total binding energy. The binding free energy (Δ*G*_bind_) of the protein–ligand complex is defined as

$$\Delta G_{\mathrm{bind}}=G_{\mathrm{complex}}-G_{\mathrm{protein}}-G_{\mathrm{ligand}}$$

Here, *G*_complex_ represents the free energy of protein–ligand complex, *G*_protein_ represents the free energy of the unbound protein, and *G*_ligand_ represents the free energy of the unbound ligand.^67^

## 3 Results

### 3.1 Molecular Docking and Visualization of Protein Ligand Interaction

Molecular docking is a widely used method for exploring how a ligand interacts with a receptor. It helps predict the binding pose of ligands within the binding site of the target receptor and estimates the binding affinity based on calculated energies. In this study, molecular docking was performed to assess the interaction of BPA analogs with LXRβ, alongside BPA itself and the reference compound G58. Typically, protein–ligand complexes with lower binding energy values indicate stronger binding affinity. Based on this criterion, the top 10 BPA analogs with the lowest binding energies were selected for further analysis, along with BPA and G58 for comparison. The RMSD between the crystallographic pose of G58 and its top-scoring redocked conformation (lowest energy pose) was calculated as 0.300 Å, indicating that the docking protocol was both accurate and reliable. The binding energy of G58 was predicted to be −12.64 kcal/mol. It interacts with the amino acid residue Leu330 via a conventional hydrogen bond. The residues Thr272 and Ala343 are involved in carbon–hydrogen bonding, while Phe268, Ile353, and Leu442 participate in alkyl interactions. Leu274, Phe329, and Phe349 are involved in pi–pi interactions. Ala275 and Leu345 interact through pi–sigma bonding, with Leu345 also forming alkyl interactions. Additionally, Met312 and Trp457 participate in pi–sulfur bonding (Figure 1A). In comparison, the binding energy of BPA was predicted to be −8.17 kcal/mol. It interacts with Ser278 via a carbon–hydrogen bond, while Ala275 and Met312 are involved in pi–alkyl interactions. Phe329 and Phe349 participate in pi–pi interactions (Figure 1B).

**Figure 1:**
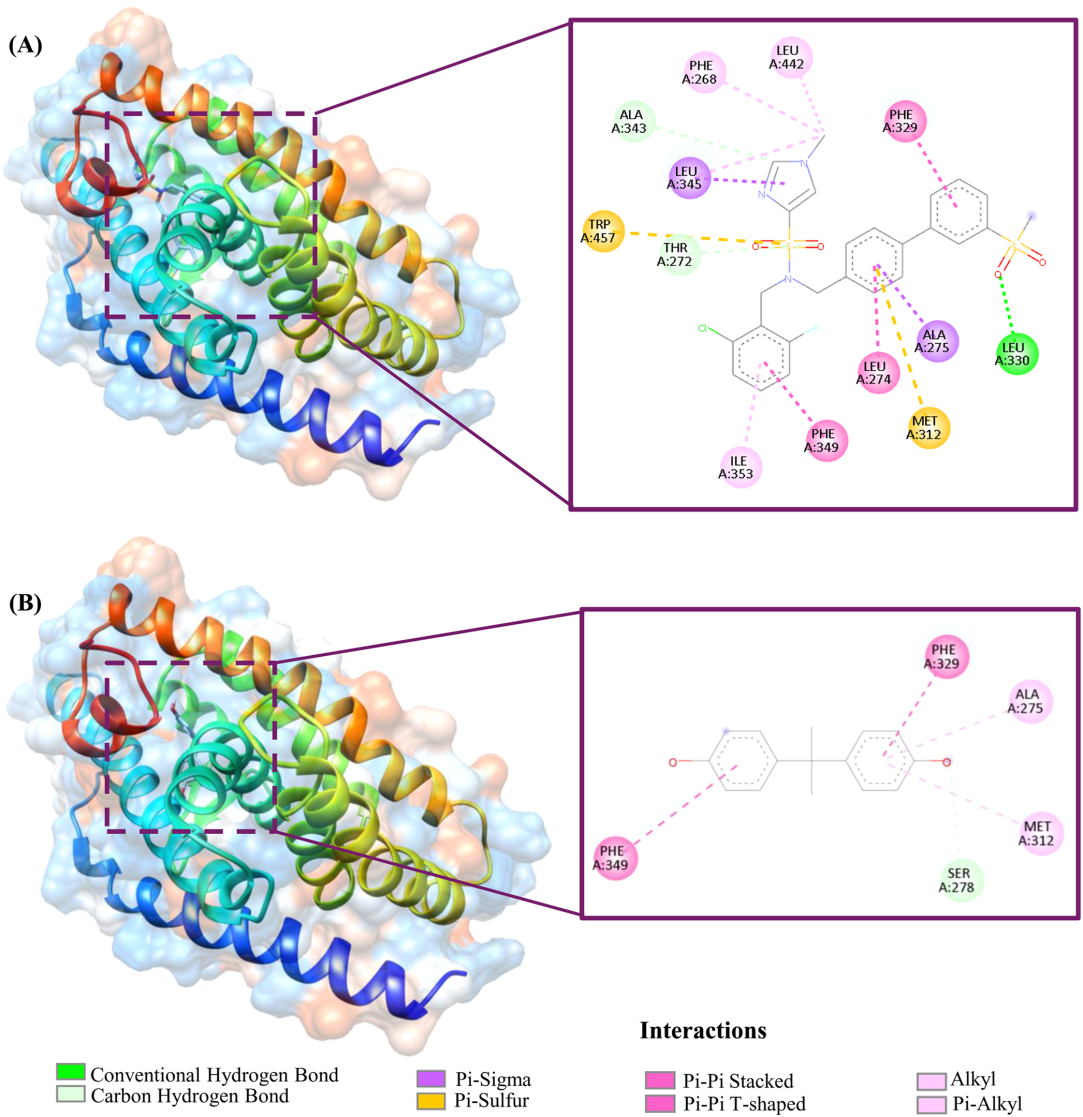
Interaction analysis of G58 and Bisphenol A with LXRβ, highlighting key interacting amino acid residues. The complexes were obtained by redocking the cocrystallized ligand G58 with its receptor LXRβ and docking BPA with LXRβ. (A) Interaction of G58. (B) Interaction of BPA, illustrating different types of molecular interactions.

Bisphenol PH (BPPH) forms conventional hydrogen bonds with Ser278 and His435, pi–sigma interactions with Ala275 and Leu345, pi–alkyl interaction with Leu274, both pi–sulfur and pi–alkyl interactions with Met312, and a pi–pi interaction with Phe329. The predicted binding energy is −12.73 kcal/mol (Figure 2A). Bisphenol P (BPP) shows pi–alkyl interactions with Ala275 and Leu442, pi–sulfur and pi–alkyl bonding with Met312, a pi–pi stacked interaction with Phe329, and a pi–sigma interaction with Leu345. The predicted binding energy is −10.68 kcal/mol (Figure 2B). Pergafast201 interacts with the amino acid Ser278 through a conventional hydrogen bond. Phe268, Ala275, Ile277, Leu330, and Leu442 are involved in alkyl interactions. Leu345 participates in both pi–sigma and alkyl bonding. Arg319 forms a pi–cation interaction, and Trp457 is involved through pi–sulfur bonding. The predicted binding energy is −10.27 kcal/mol (Figure 2C). 4-((4-(Benzyloxy)­phenyl)­sulfonyl)­phenol (BPSP) interacts with Leu274 and His435 via conventional hydrogen bonds, Thr272 and Leu345 through pi–sigma bonding, and Met312 through both pi–sulfur and pi–alkyl interactions. Phe329 and Phe349 are involved in pi–pi interactions. The binding energy is −10.11 kcal/mol (Figure 2D). Bisxylenol A (BXA) interacts with Phe329 through pi–donor hydrogen bonding, pi–sigma, and pi–pi stacked interactions. It also interacts with Phe271 via pi–sigma bonding, Ala275 via pi–alkyl, Leu274 via pi–pi stacking, and Leu345 via pi–sigma bonding. The binding energy is −9.81 kcal/mol (Figure 2E). Bisphenol Z (BPZ) forms pi–alkyl interactions with Ala275, Leu345, and Ile353; pi–sulfur bonding with Met312; and a pi–pi stacked interaction with Phe329. The predicted binding energy is −9.56 kcal/mol (Figure 2F). Bis-o-cresol A interacts with Ala275 and Leu345 via pi–sigma bonding. It also interacts with Phe329 through both pi–sigma and pi–pi stacked interactions, and with Met312 through pi–sulfur bonding. The binding energy is −9.44 kcal/mol (Figure 2G). Bisphenol AF (BPAF) interacts with Ala275 via a pi–alkyl bond, with Met312 through pi–sulfur and alkyl interactions, with Phe340 via an alkyl bond, and with Leu345 through both alkyl and pi–alkyl bonds. Phe329 is involved in a pi–pi stacked interaction. The binding energy is −9.22 kcal/mol (Figure 2H). 2,2′-Diallyl-4,4′-sulfonyldiphenol interacts with Leu274 through a conventional hydrogen bond. It also forms an alkyl interaction with Phe268, alkyl and pi–alkyl interactions with Leu345, and a pi–alkyl interaction with Ala275. Additionally, Phe329 is involved through pi–pi stacked and alkyl interactions. The predicted binding energy is −8.89 kcal/mol (Figure 2I). Bisphenol AP (BPAP) interacts with Leu274 through both a conventional hydrogen bond and a pi–alkyl interaction. It also interacts with Thr272 via van der Waals forces, and with Ala275, Leu313, and Ile353 through pi–alkyl interactions. Furthermore, it forms pi–pi stacked interactions with Phe271 and Phe329. The binding energy is −8.89 kcal/mol (Figure 2J). Details of all the selected BPA analogs, their binding free energies with LXRβ, and the key LXRβ amino acid residues involved in the interactions are summarized in Table 1.

**Figure 2:**
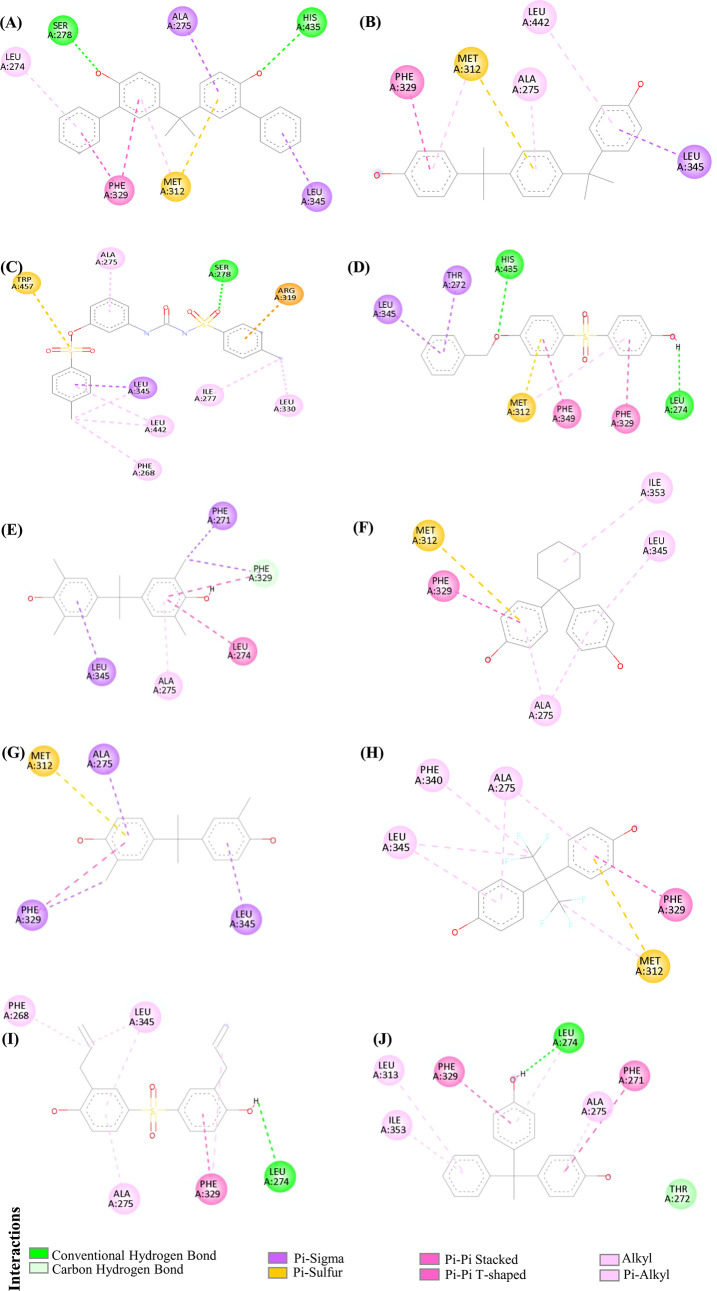
2D representations of the molecular interactions between the top 10 BPA analogs and LXRβ. The figures illustrate key amino acid residues contributing to protein–ligand interactions: (A) BPPH, (B) BPP, (C) Pergafast201, (D) BPSP, (E) BXA, (F) BPZ, (G) Bis-o-cresol A, (H) BPAF, (I) 2,2′-Diallyl-4,4′-sulfonyldiphenol , and (J) BPAP.

**Table 1:** Comparative Binding Free Energies of BPA Analogs with LXRβ, Including Their CAS and PubChem Identifiers, with Highlighted Amino Acid Intermolecular Contacts^a^

S.N.	Compound name	CAS No.	PubChem CID	Docking score (kcal/mol)	Amino acid contacts
1.	N-(2-chloro-6-fluorobenzyl)-1-methyl-N-{[3′-(methylsulfonyl)biphenyl-4-yl]methyl}-1H-imidazole-4-sulfonamide or G58	1221277-91-3	45100506	–12.64	Phe268, Thr272, Leu274, Ala275, Met312, Phe329, Leu330, Ala343, Leu345, Phe349, Ile353, Leu442, Trp457
2.	Bisphenol A (BPA)	80-05-7	6623	–8.174	Ala275, Ser278, Met312, Phe329, Phe349
3.	2,2-Bis(2-hydroxy-5-biphenylyl)propane (Bisphenol PH or BPPH)	24038-68-4	13059052	–12.73	Leu274, Ala275, Ser278, Met312, Phe329, Leu345, His435
4.	Bisphenol P (BPP)	2167-51-3	630355	–10.68	Ala275, Met312, Phe329, Leu345, Leu442
5.	3-(3-Tosylureido)phenyl p-toluenesulfonate or (Pergafast 201)	232938-43-1	22035425	–10.27	Phe268, Thr272, Leu274, Ala275, Met312, Phe329, Leu330, Ala343, Leu345, Phe349, Ile353, Leu442, Trp457
6.	4-Benzyloxyphenyl 4-hydroxyphenyl sulfone or 4-((4-(Benzyloxy)phenyl)sulfonyl)phenol (BPSP)	63134-33-8	113063	–10.11	Thr272, Leu274, Met312, Phe329, Leu345, Phe349, His435
7.	2,2-Bis(4-hydroxy-3,5-dimethylphenyl)propane (Bisxylenol A or BXA)	5613-46-7	79717	–9.812	Phe271, Leu274, Ala275, Phe329, Leu345
8.	Bisphenol Z (BPZ)	843-55-0	232446	–9.561	Ala275, Met312, Phe329, Leu345, Ile353
9.	2,2-Bis(4-hydroxy-3-methylphenyl)propane or Bis-o-cresol A	79-97-0	6620	–9.448	Ala275, Met312, Phe329, Leu345
10.	Bisphenol AF (BPAF)	1478-61-1	73864	–9.224	Ala275, Met312, Phe329, Phe340, Leu345
11.	2,2’-Diallyl-4,4’-sulfonyldiphenol	41481-66-7	833466	–8.982	Phe268, Leu274, Ala275, Phe329, Leu345
12.	Bisphenol AP (BPAP)	1571-75-1	623849	–8.897	Phe271, Thr272, Leu274, Ala275, Leu313, Phe329, Ile353
13.	4,4″-Bis(p-tolylsulfonylureido)-diphenylmethane or Benzenesulfonamide, N,N’-(methylenebis(4,1-phenyleneiminocarbonyl))bis(4-methyl (BTUM)	151882-81-4	3596056	–8.89	Val232, Leu236, Asn239, Arg318, Arg319, Asn321, His322, Ser361, Asp367
14.	4-((4-Isopropoxyphenyl)sulfonyl)phenol	95235-30-6	9904141	–8.604	Ser278, Met312, Phe329, Phe349, Val439, His435, Leu442, Leu449, Trp457
15.	4-((4-(Allyloxy)phenyl)sulfonyl)phenol	97042-18-7	2054598	–8.393	Leu274, Ala275, Met312, Phe329, Phe349, His435, Leu449, Leu453
16.	2,4′-Dihydroxydiphenyl sulfone (2,4-BPS)	5397-34-2	79381	–8.236	Ser278, Met312, Thr316, Phe329, Phe349
17.	Bisphenol B (BPB)	77-40-7	66166	–8.144	Ala275, Ser278, Met312, Phe329, Leu345
18.	Bisphenol A bis(diphenyl phosphate)	5945-33-5	9874825	–8.073	Arg318, Arg319, His322, Ser361
19.	Bisphenol E (BPE)	2081-80-5	608116	–7.891	Leu274, Ala275, Met312, Phe329, Leu345
20.	2,2′-Bisphenol F(2,2′-BPF)	2467-02-9	75575	–7.753	Ala275, Thr316, Phe329
21.	Methyl bis(4-hydroxyphenyl)acetate (MBHA)	5129-00-0	78805	–7.729	Leu274, Ala275, Ser278, Met312, Phe329, Leu345
22.	Bis[2-(4-hydroxyphenylthio)ethoxy]methane	93589-69-6	3086375	–7.589	Leu274, Ala275, Met312, Phe329, Leu330, Leu345, Trp457
23.	Bisphenol F (BPF)	620-92-8	12111	–7.559	Phe329, Leu345

a G58 and BPA were used as reference compounds.

### 3.2 Prediction and Analysis of Pharmacokinetic Properties and Toxicity

The top 10 BPA analogs, alongside reference compounds BPA and G58, underwent pkCSM-based pharmacokinetic and toxicity predictions. These predictions show strong consistency with key experimental data for BPA, including the experimental confirmation of its negative Ames test and validation of its blood–brain barrier penetration mechanism, supporting the reliability of the applied approaches. Absorption metrics comprised water solubility and intestinal absorption capacity. The water solubility and intestinal absorption of the BPA analogs were predicted to range from −5.961 to −3.855 log mol/L and 86.155% to 96.746%, respectively. In comparison, G58 and BPA showed predicted water solubilities of −3.239 and −3.641 log mol/L, and intestinal absorptions of 96.824% and 93.259%, respectively. In the distribution prediction, blood–brain barrier (BBB) permeability and central nervous system (CNS) permeability were evaluated. For BPA analogs, the predicted BBB permeability ranged from −0.482 to 0.17 log BB, and CNS permeability ranged from −3.141 to −0.564 log PS. In comparison, the predicted BBB permeability values for G58 and BPA were −1.950 and 0.045 log BB, respectively, while their CNS permeability values were −3.089 and −1.584 log PS, respectively. Metabolism predictions assessed three parameters: CYP2D6 substrate potential, CYP3A4 substrate potential, and CYP1A2 inhibition. All evaluated BPA analogs, including reference compounds G58 and BPA, were predicted as nonsubstrates for CYP2D6 but substrates for CYP3A4. For CYP1A2 inhibition, all compounds except G58 and Pergafast201 were predicted inhibitors. Excretion analysis revealed total clearance values ranging from −0.293 to 0.895 log mL/min/kg among BPA analogs. Reference compounds showed lower clearance: −0.055 log mL/min/kg (G58) and 0.125 log mL/min/kg (BPA). Toxicity assessment evaluated AMES mutagenicity, acute oral toxicity in rats, and chronic oral toxicity in rats. Mutagenicity predictions indicated negative AMES results for all compounds except BPPH, BPP, Bis-o-cresol A, BPAP, and G58. Oral rat acute toxicity and chronic toxicity values for the BPA analogs were estimated to range from 1.925 to 3.106 mol/kg and 1.037 to 2.448 log mg/kg_bw/day, respectively. In contrast, for G58 and BPA, the predicted values were 2.673 and 2.427 mol/kg for acute toxicity, and −0.042 and 1.843 log mg/kg_bw/day for chronic toxicity, respectively (Table 2). The pharmacokinetics and toxicology predictions suggest that BPA analogs exhibit properties similar to BPA, indicating potential risks to human health and the environment.

**Table 2:** Comparative Pharmacokinetic and Toxicity Profiles of High-Affinity BPA Analogs Bound to LXRβ (Top 10) and Reference Ligands (G58, BPA)

Property	Parameter	G58	BPA	BPPH	BPP	Pergafast201	BPSP	BXA	BPZ	Bis-o-cresol A	BPAF	2,2’-Diallyl-4,4’-sulfonyldiphenol	BPAP
Absorption	Water solubility (log mol/L)	–3.239	–3.641	–4.566	–5.264	–5.961	–4.321	–4.189	–4.261	–3.855	–4.207	–4.205	–4.611
	Intestinal absorption (% Absorbed)	96.824	93.259	95.423	93.008	92.036	94.953	91.37	94.371	91.982	86.155	93.183	96.746
Distribution	BBB permeability (log BB)	–1.95	0.045	–0.188	–0.031	–0.482	0.086	0.17	–0.188	0.089	0.154	–0.181	–0.096
	CNS permeability (log PS)	–3.089	–1.584	–0.564	–1.493	–3.141	–2.141	–1.666	–1.513	–1.83	–1.733	–2.364	–1.417
Metabolism	CYP2D6 substrate (Yes/No)	No	No	No	No	No	No	No	No	No	No	No	No
	CYP3A4 substrate (Yes/No)	Yes	Yes	Yes	Yes	Yes	Yes	Yes	Yes	Yes	Yes	Yes	Yes
	CYP1A2 inhibitor (Yes/No)	No	Yes	Yes	Yes	No	Yes	Yes	Yes	Yes	Yes	Yes	Yes
Excretion	Total clearance (log mL/min/kg)	–0.055	0.125	0.228	0.094	0.593	0.626	0.895	0.073	0.128	–0.293	0.695	0.095
Toxicity	AMES toxicity (Yes/No)	Yes	No	Yes	Yes	No	No	No	No	Yes	No	No	Yes
	Oral rat acute toxicity (LD50) (mol/kg)	2.673	2.427	3.106	2.106	2.112	2.114	2.41	2.126	2.408	2.66	1.925	2.273
	Oral rat chronic toxicity (LOAEL) (logmg/kg_bw/day)	–0.042	1.843	1.391	1.51	2.448	1.195	1.528	2.006	1.533	1.037	2.12	1.434

### 3.3 MD Simulation for Exploring Structural Stability, Flexibility, and Binding Interaction

To evaluate the dynamic behavior of LXRβ and its conformational changes upon interaction with BPA analogs, MD simulations were performed. Protein dynamics were characterized using various parameters such as conformational stability (RMSD), residue flexibility (RMSF), molecular compactness (Rg), surface exposure (SASA), intermolecular interactions (HB), essential motions (PCA), and thermodynamic stability (FEL). G58 and BPA were included as reference compounds. The results are summarized in the following subsections and Table S1 (Supporting Information).

#### 3.3.1 Conformational Stability

The conformational stability of LXRβ was assessed by measuring the RMSD during MD simulation. Lower RMSD values indicate greater structural stability, while higher values suggest increased movement or change. In this analysis, RMSD was calculated over a 100 ns period, reflecting how much the structure deviates from its initial conformation throughout the simulation. The RMSD plot of backbone Cα atoms showed that both LXRβ and all its complexes maintained low RMSD values, indicating stable conformations. The average RMSD of LXRβ was calculated as 0.11 nm. However, the RMSD values of LXRβ-G58, LXRβ-BPA, LXRβ-BPPH, LXRβ-BPP, LXRβ-Pergafst201, LXRβ-BPSP, LXRβ-BXA, LXRβ-BPZ, LXRβ-Bis-o-cresol A, LXRβ-BPAF, LXRβ-2,2’-Diallyl-4,4’-sulfonyldiphenol, and LXRβ-BPAP complexes were 0.16, 0.14, 0.16, 0.12, 0.11, 0.11, 0.11, 0.12, 0.11, 0.12, 0.11, and 0.11 nm, respectively. All systems demonstrated stability over the course of the simulation, resulting in the formation of stable complexes (Figure 3A).

**Figure 3:**
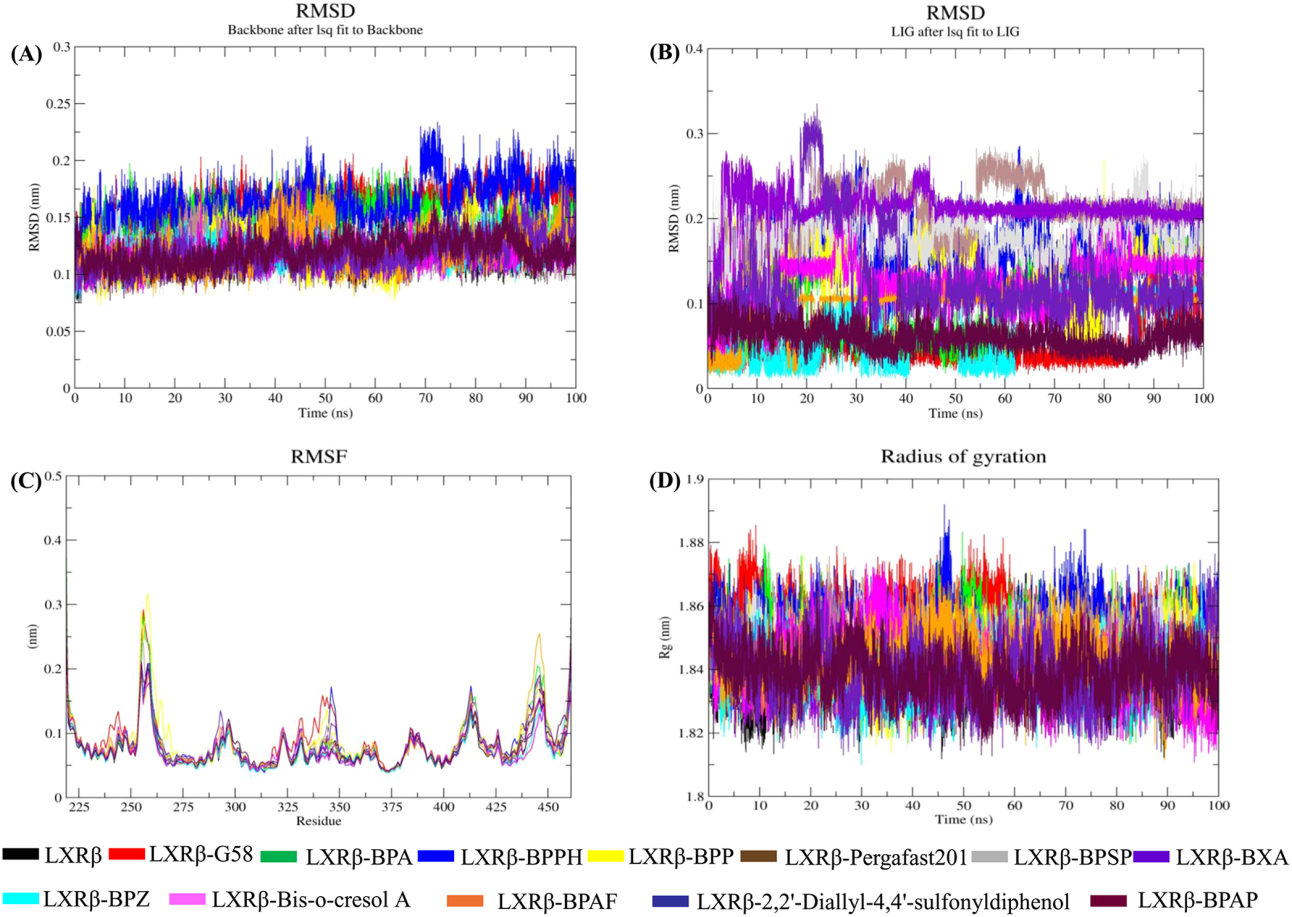
Stability and structural analysis of LXRβ complexes. (A) RMSD plot of LXRβ, LXRβ-G58, LXRβ-BPA, and LXRβ-BPA analog complexes. (B) Ligand RMSD plot of G58, BPA, and BPA analogs. (C) RMSF plot of LXRβ, LXRβ-G58, LXRβ-BPA, and LXRβ-BPA analog complexes, showing residue-level flexibility. (D) Radius of gyration (Rg) plot of LXRβ, LXRβ-G58, LXRβ-BPA, and LXRβ-BPA analog complexes over 100 ns of simulation, indicating compactness.

Furthermore, the structural stability of the BPA analogs, along with G58 and BPA, within the LXRβ binding pocket was evaluated by calculating ligand RMSD over the simulation time. All ligands exhibited RMSD values consistently below 0.3 nm, except for LXRβ-BXA, indicating stable binding within the pocket. Although LXRβ-BXA showed a higher RMSD initially, it stabilized after approximately 30 ns. The average ligand RMSD values for LXRβ-G58, LXRβ-BPA, LXRβ-BPPH, LXRβ-BPP, LXRβ-Pergafast 201, LXRβ-BPSP, LXRβ-BXA, LXRβ-BPZ, LXRβ-Bis-o-cresol A, LXRβ-BPAF, LXRβ- 2,2’-Diallyl-4,4’-sulfonyldiphenol, and LXRβ-BPAP were 0.05, 0.10, 0.16, 0.12, 0.20, 0.18, 0.21, 0.07, 0.12, 0.09, 0.12, and 0.06 nm, respectively (Figure 3B).

#### 3.3.2 Flexibility and Residual Mobility

RMSF analysis provides insight into protein characteristics by highlighting the flexibility of individual residues. Accordingly, the RMSF of LXRβ and its ligand-bound complexes was analyzed over a 100 ns simulation. The average RMSF value of LXRβ was calculated as 0.07 nm. However, the RMSF values of LXRβ-G58, LXRβ-BPA, LXRβ-BPPH, LXRβ-BPP, LXRβ-Pergafst201, LXRβ-BPSP, LXRβ-BXA, LXRβ-BPZ, LXRβ-Bis-o-cresol A, LXRβ-BPAF, LXRβ-2,2’-Diallyl-4,4’-sulfonyldiphenol, and LXRβ-BPAP complexes were 0.08, 0.08, 0.08, 0.08, 0.08, 0.08, 0.07, 0.07, 0.07, 0.08, 0.08, and 0.07 nm, respectively (Figure 3C).

#### 3.3.3 Compactness

To evaluate the changes in protein compactness, stability, and folding over time, Rg was analyzed. This parameter, which reflects structural compactness, was calculated for LXRβ and its complexes. The average Rg value for LXRβ was calculated as 1.83 nm. Furthermore, the average Rg values of LXRβ-G58, LXRβ-BPA, LXRβ-BPPH, LXRβ-BPP, LXRβ-Pergafst201, LXRβ-BPSP, LXRβ-BXA, LXRβ-BPZ, LXRβ-Bis-o-cresol A, LXRβ-BPAF, LXRβ-2,2’-Diallyl-4,4’-sulfonyldiphenol, and LXRβ-BPAP complexes were 1.85, 1.84, 1.85, 1.84, 1.84, 1.84, 1.83, 1.83, 1.84, 1.84, 1.83, and 1.83 nm, respectively (Figure 3D).

#### 3.3.4 Solvent-Accessible Surface Area (SASA)

Solvent-accessible surface area (SASA) analysis was performed over the 100 ns simulation time frame to evaluate the influence of ligand binding on the LXRβ surface exposure to the solvent. The average SASA values for the LXRβ, LXRβ-G58, LXRβ-BPA, LXRβ-BPPH, LXRβ-BPP, LXRβ-Pergafast201, LXRβ-BPSP, LXRβ-BXA, LXRβ-BPZ, LXRβ-Bis-o-cresol A, LXRβ-BPAF, LXRβ- 2,2’-Diallyl-4,4’-sulfonyldiphenol, and LXRβ-BPAP complexes were calculated as 132.07, 136.07, 132.92, 133.65, 132.94, 132.48, 132.48, 131.13, 131.61, 130.94, 132.56, 132.54, and 130.83 nm^2^, respectively. The SASA value of the LXRβ–G58 complex was higher than that of LXRβ–BPA and the other complexes. Still, all systems showed a similar overall pattern, which suggests that ligand binding caused only small changes in the protein’s surface exposure (Figure S2).

#### 3.3.5 Hydrogen Bond Interaction Profiling

Protein–ligand stabilization involves various interactions; to evaluate the role of hydrogen bonding specifically, analysis was conducted over a 100 ns simulation period. The results are shown in Figures 4 and 5. The reference compounds G58 (Figure 4A) and BPA (Figure 4B) formed 0–4 and 0–3 hydrogen bonds with LXRβ, respectively. Similarly, the BPA analogs such as BPPH (Figure 5A), BPP (Figure 5B), Pergafast201 (Figure 5C), BPSP (Figure 5D), BXA (Figure 5E), BPZ (Figure 5F), Bis-o-cresol A (Figure 5G), BPAF (Figure 5H), 2,2’-Diallyl-4,4’-sulfonyldiphenol (Figure 5I), and BPAP (Figure 5J) exhibited hydrogen bond counts in the range of 0–3. Based on this analysis, the BPA analogs displayed hydrogen bonding patterns with LXRβ that were comparable to those of G58 and BPA, suggesting stable and consistent interactions within the LXRβ binding cavity.

**Figure 4:**
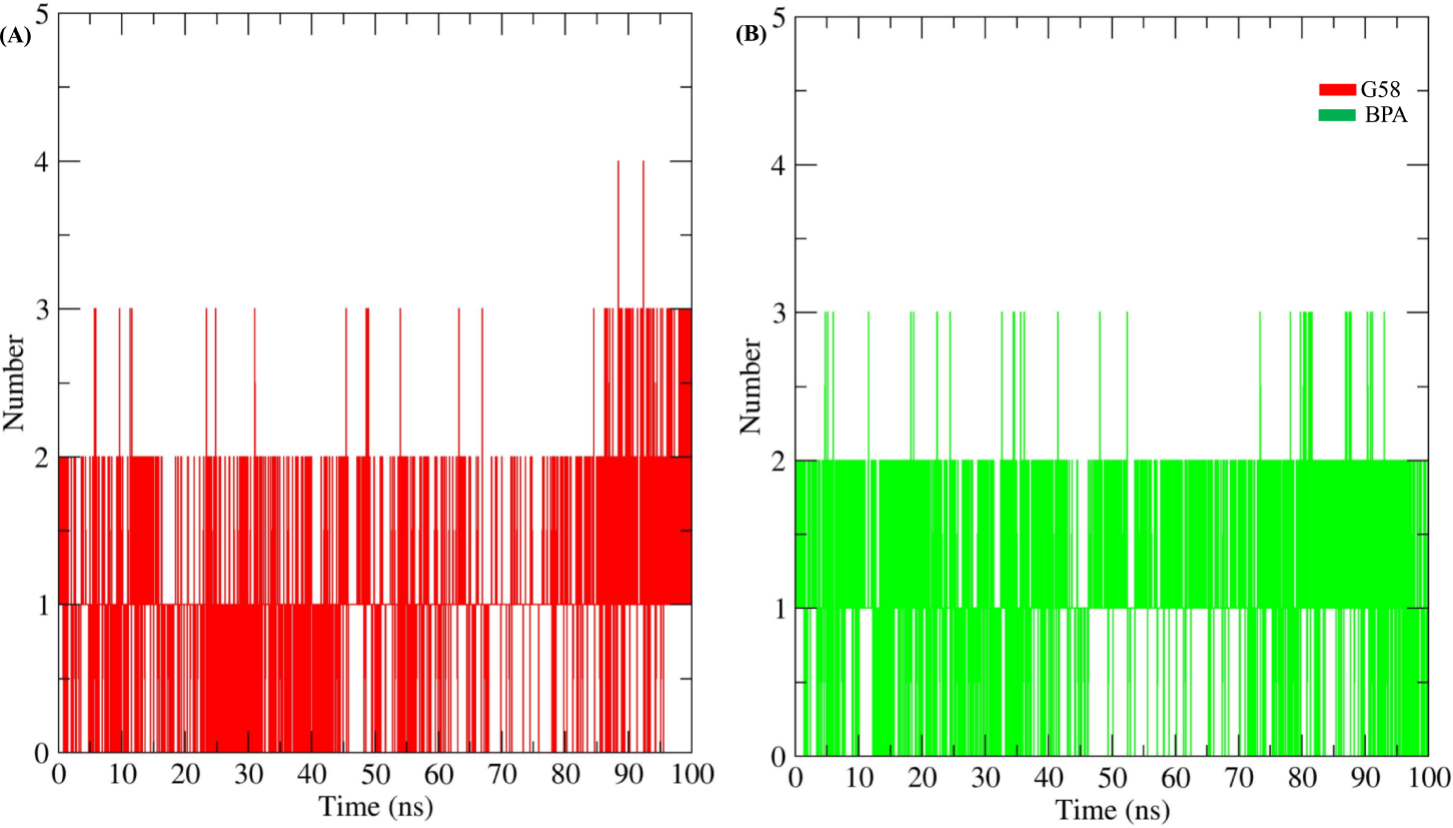
Hydrogen bond analysis of ligand interactions with LXRβ. (A) G58 and (B) BPA showing the number of hydrogen bonds formed with LXRβ over 100 ns of simulation time.

**Figure 5:**
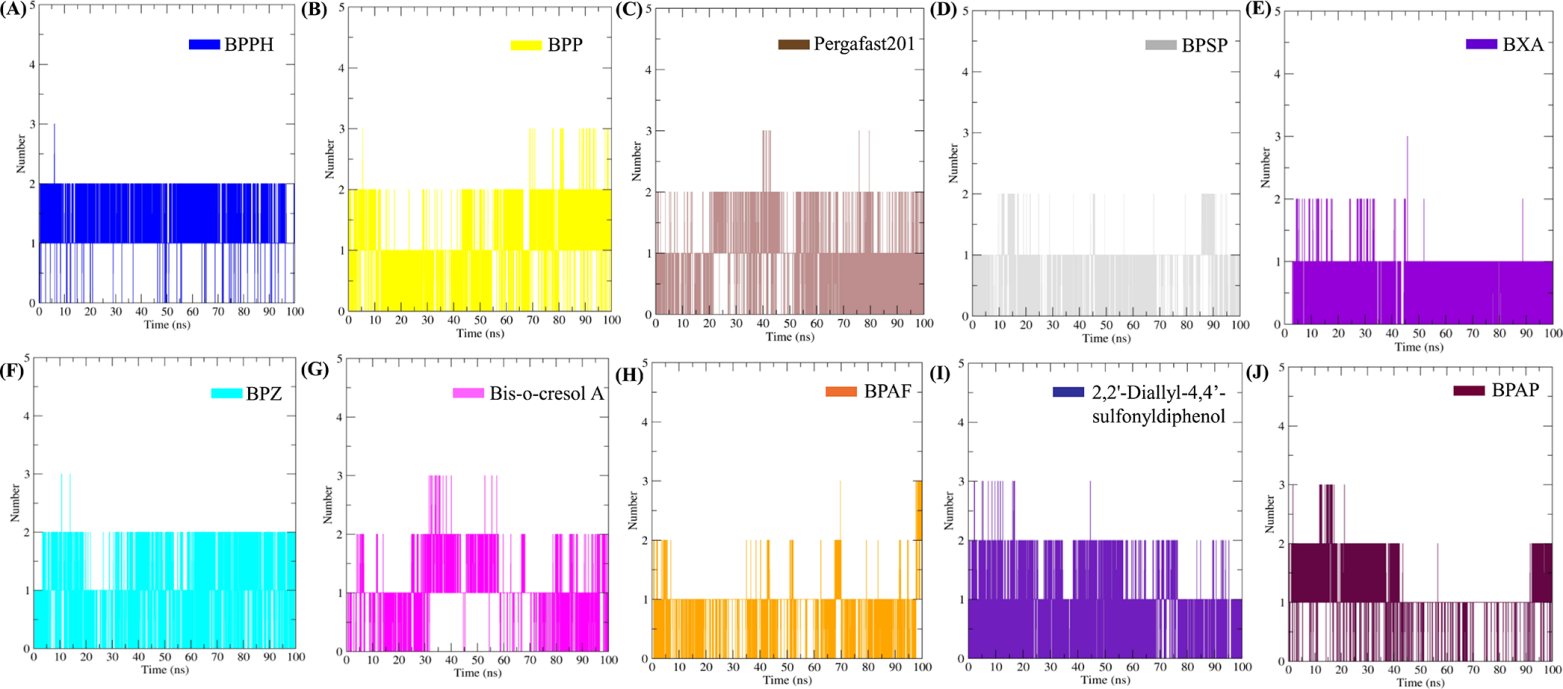
Hydrogen bond analysis of ligand interactions with LXRβ. (A) BPPH, (B) BPP, (C) Pergafast201, (D) BPSP, (E) BXA, (F) BPZ, (G) Bis-o-cresol A, (H) BPAF, (I) 2,2′-Diallyl-4,4′-sulfonyldiphenol, and (J) BPAP showing the number of hydrogen bonds formed with LXRβ over 100 ns of simulation time.

#### 3.3.6 Essential Dynamics

To better understand the conformational behavior of the protein, essential dynamics analysis was carried out using PCA. The major structural motions are typically captured by the first few eigenvectors. Therefore, the top 50 eigenvectors were considered to explore overall structural shifts. To gain deeper insights into ligand-induced motions, percentage-wise correlated movements were calculated from the first ten eigenvectors, providing a clearer view of the key dynamic changes. LXRβ, LXRβ-G58, LXRβ-BPA, LXRβ-BPPH, LXRβ-BPP, LXRβ-Pergafst201, LXRβ-BPSP, LXRβ-BXA, LXRβ-BPZ, LXRβ-Bis-o-cresol A, LXRβ-BPAF, LXRβ- 2,2’-Diallyl-4,4’-sulfonyldiphenol, and LXRβ-BPAP showed 62.05%, 72.21%, 69.76%, 67.01%, 73.33%, 68.96%, 64.31%, 62.14%, 60.90%, 62.02%, 69.83%, 66.30%, 63.05% correlated motions, respectively. LXRβ-BPZ, LXRβ-Bis-o-cresol A, LXRβ, and LXRβ-BXA were predicted to exhibit the lowest levels of motion (Figure 6A). Since the first few eigenvectors typically capture the overall dynamics of a protein, the first two were selected and plotted in phase space. The clusters corresponding to LXRβ-BPZ, LXRβ–Bis-o-cresol A, LXRβ, and LXRβ-BXA appeared to be the most stable, showing lower correlated motions compared to the other complexes (Figure 6B).

**Figure 6:**
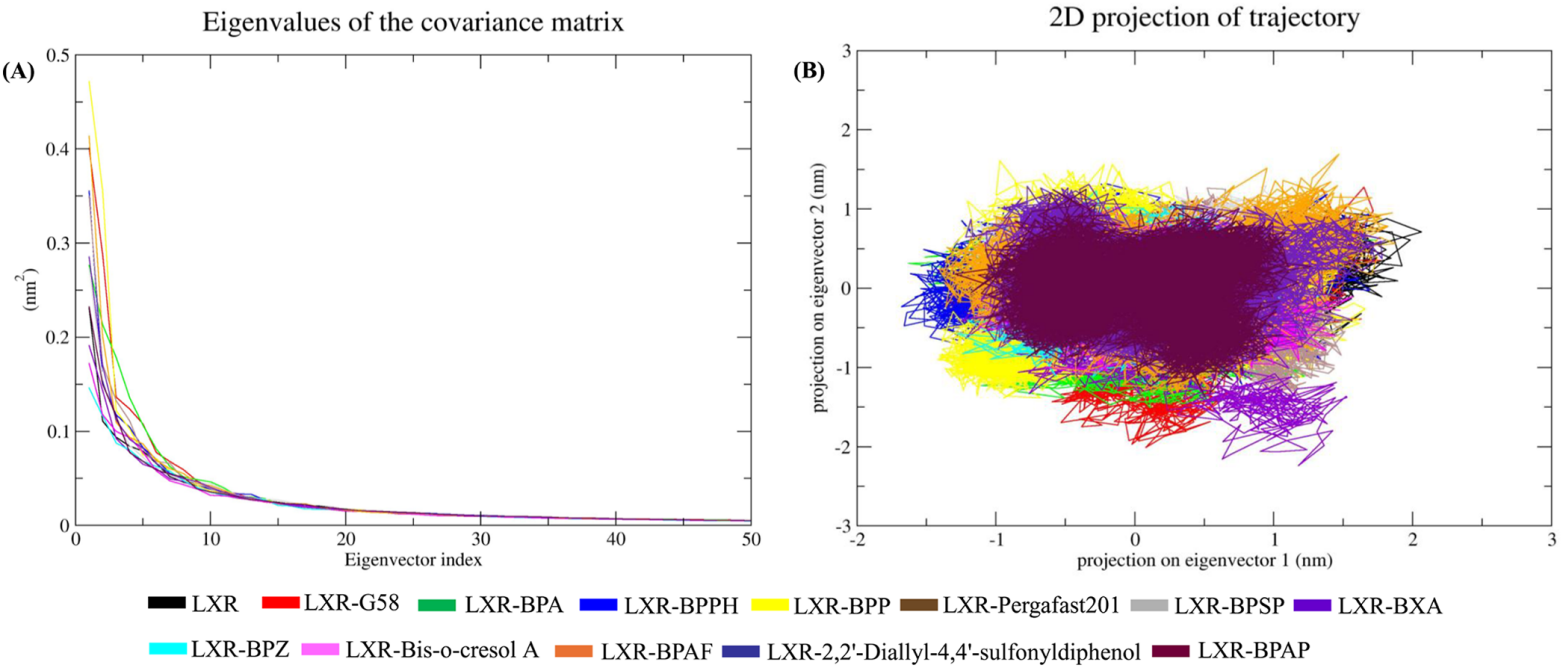
Essential dynamics of LXRβ and its complexes based on principal component analysis. (A) Eigenvalue distribution plot showing the contribution of the first 50 eigenvectors to the overall motion during the 100 ns simulation. (B) Projection of the first two principal components (PC1 vs PC2), illustrating the collective motion of LXRβ in the apo form and ligand-bound complexes.

#### 3.3.7 Gibbs Free Energy Landscape (FEL)

To understand the thermodynamic nature of the system, the Gibbs free energy landscape (FEL) was analyzed to identify energy minima, conformational flexibility, and stability at the atomic level. The first two principal components were used for FEL analysis of each system, as shown in the Supporting Information Figure S3 and S4. In the figures, blue areas indicate the lowest energy states, while red areas represent the highest energy states, measured in kJ mol^–1^ (Figure S3). Energy values ranging from 0 to 13, 0–12.3, 0–12.1, 0–11.9, 0–12.1, 0–12.4, 0–11.3, 0–13.5, 0–12.5, 0–11.8, 0–12.7, 0–11.7, and 0–11.7 kJ mol^–1^ were predicted for LXRβ, LXRβ-G58, LXRβ-BPA, LXRβ-BPPH, LXRβ-BPP, LXRβ-Pergafst201, LXRβ-BPSP, LXRβ-BXA, LXRβ-BPZ, LXRβ-Bis-o-cresol A, LXRβ-BPAF, LXRβ-2,2’-Diallyl-4,4’-sulfonyldiphenol, and LXRβ-BPAP, respectively. LXRβ-BPPH, LXRβ-BPSP, LXRβ- Bis-o-cresol A, LXRβ- 2,2’-Diallyl-4,4’-sulfonyldiphenol, and LXRβ-BPAP showed slightly lower values (Figure S4). During the simulation, these compounds underwent conformational transitions that corresponded to energetically favorable states.

### 3.4 MM/PBSA Binding Energy Calculations

Binding free energies of the simulated complexes were estimated using the MM-PBSA approach to validate their binding affinities. The calculations were performed using trajectory frames from the final 10 ns of the molecular dynamics simulations. The calculated total binding free energy for LXRβ-G58, LXRβ-BPA, LXRβ-BPPH, LXRβ-BPP, LXRβ-Pergafst201, LXRβ-BPSP, LXRβ-BXA, LXRβ-BPZ, LXRβ-Bis-o-cresol A, LXRβ-BPAF, LXRβ- 2,2’-Diallyl-4,4’-sulfonyldiphenol, and LXRβ-BPAP complexes were −27.81, −18.21, −32.81, −28.99, −23.38, −19.92, −24.12, −23.70, −23.38, −23.00, −17.54, and −22.86 kcal/mol, respectively. BPPH and BPP showed higher binding affinity compared to G58. However, all selected BPA analogs exhibited better binding affinity with LXRβ than BPA, except for 2,2’-Diallyl-4,4’-sulfonyldiphenol. Key energy contributions governing ligand binding (van der Waals, electrostatic, Poisson–Boltzmann, polar solvation, gas-phase, and solvation free energy) are presented in Table 3.

**Table 3:** MM-PBSA Binding Free Energy Calculations of BPA Analogs with LXRβ, Including G58 and BPA^a^ ^b^

S.N.	Compounds	Δ_Vdw_	Δ*E*_EL_	Δ*E*_PB_	Δ*E*_NPOLAR_	Δ*G*_GAS_	Δ*G*_Sol_	Δ_Total_
1.	G58	–59.33	75.22	–38.09	–5.60	15.89	–43.69	–27.81
2.	BPA	–26.87	–8.55	20.59	–3.38	–35.42	17.21	–18.21
3.	BPPH	–44.31	–14.38	30.72	–4.85	–58.69	25.88	–32.81
4.	BPP	–42.56	–11.19	29.47	–4.71	–53.75	24.76	–28.99
5.	Pergafast 201	–60.22	–15.18	57.41	–5.39	–75.41	52.02	–23.38
6.	BPSP	–42.74	–10.19	37.35	–4.34	–52.93	33.01	–19.92
7.	BXA	–40.12	–7.08	27.05	–3.97	–47.20	23.08	–24.12
8.	BPZ	–34.69	–9.23	23.89	–3.68	–43.91	20.21	–23.70
9.	Bis-o-cresol A	–36.68	–11.95	28.78	–3.53	–48.63	25.25	–23.38
10.	BPAF	–33.42	–10.72	24.72	–3.57	–44.14	21.15	–23.00
11.	2,2’-Diallyl-4,4’-sulfonyldiphenol	–41.40	–10.98	39.30	–4.46	–52.38	34.84	–17.54
12.	BPAP	–38.49	–13.50	32.94	–3.81	–51.99	29.14	–22.86

a Predicted values include van der Waals energy, electrostatic energy, Poisson–Boltzmann energy, polar solvation energy, gas-phase free energy, solvation free energy, and total binding energy (kcal/mol).

b ΔVdw: van der Waals energy; Δ*E*_EL_: electrostatic energy; Δ*E*_PB_: Poisson–Boltzmann energy; Δ*E*_NPOLAR_: polar solvation energy; Δ*G*_gas_: gas-phase free energy; Δ*G*_Sol_: solvation free energy; Δ_Total_: total binding energy.

## 4 Discussion

BPA is a well-documented hazardous chemical that poses risks to human health, animals, and the environment.^1,15^ Several research projects have been completed, and many are ongoing to evaluate the health risks of BPA under different scenarios, in line with project objectives. In recent years, researchers have also assessed the risks associated with BPA analogs, which are frequently used in the manufacturing of various products as substitutes for BPA. However, several studies have raised concerns about their safety, as some BPA analogs have been reported to exhibit toxic effects similar to those of BPA.,^68^ A recent study in rats showed that oral exposure to BPA disrupts the gut-liver-hormone axis, affecting multiple organs, altering serum biochemistry, gut microbiota, and short-chain fatty acids. It also changed key metabolites and caused potential systemic toxicity.^69^ Guo et al. reported that bisphenol analogs disrupt hepatic metabolism across multiple species, inducing liver toxicity through mechanisms such as oxidative stress, lipid accumulation, and inflammation. However, species-specific differences in metabolic capacity, clearance rates, and hormonal regulation complicate the extrapolation of animal data to humans. Therefore, targeted research is needed to improve risk assessment.^70^

Liver X receptors (LXRs), originally discovered in the liver but expressed in multiple organs, are nuclear receptors that play key roles in controlling lipid metabolism and inflammatory responses. They exist in two forms: Liver X receptor-alpha (LXRα) and Liver X receptor-beta (LXRβ), which share about 77% sequence similarity.^38,71,72^ Among the two, LXRβ is more prominently expressed in the brain.^38,73^ Studies have shown that BPA can activate LXR, leading to hypoglycemia, disruption of glucose metabolism, and impaired liver function.^24,74^ The mechanism by which BPA interacts with LXR is not yet well understood. A computational study demonstrated the interaction of BPA with both mouse and human LXRs, predicting that BPA binds tightly within the ligand-binding pocket.^24^ Therefore, the presence of BPA analogs in the environment, along with existing toxicity data, emphasizes the need to investigate their interactions with LXRs to support health risk assessment.^17,24,44^

In this study, we sought to explore the molecular basis of BPA analogs’ interactions with LXRβ. Molecular docking revealed that several BPA analogs exhibit greater predicted binding affinity for LXRβ than BPA itself.^75,76^ This finding highlights their stronger potential to modulate receptor activity. Our analysis also identified the key amino acid residues involved in these interactions. The docking results, supported by molecular dynamics simulations, free energy landscape (FEL) analysis, and binding energy calculations, provided further insight into the stability and strength of these interactions.^75,76^ In addition, pharmacokinetics and pharmacodynamics evaluations indicated the potential bioactivity and systemic behavior of these compounds.^76^ Collectively, these findings highlight the importance of characterizing BPA analogs individually, as their structural variations may result in differential binding behaviors and toxicological outcomes.^21,23^

Molecular docking was used to explore how BPA analogs interact with LXRβ, particularly in comparison to BPA and G58. Subsequent to the structural analysis of the protein–ligand complexes, the top 10 BPA analogs, along with BPA and G58, were further evaluated for their pharmacokinetic and pharmacodynamic properties—an essential step in assessing the potential of candidate molecules through computational methods. Most of the selected BPA analogs showed predicted stronger binding affinity to LXRβ than BPA, except for BPB, Bisphenol A bis­(diphenyl phosphate), BPE, 2,2′-Bisphenol F (2,2′-BPF), MBHA, Bis­[2-(4-hydroxyphenylthio)­ethoxy]­methane, and BPF. Interestingly, the top 10 BPA analogs exhibited comparable ADME and toxicity profiles, raising concerns about their safety in industrial applications.,^68^ Experimental studies report negative Ames toxicity for BPA, which is consistent with our computational predictions.^77^ Furthermore, experimental models confirm that BPA can cross the blood–brain barrier and can also inhibit protective efflux transporters like BCRP, potentially disrupting its function.^78^ However, some of the top-screened compounds showed positive Ames toxicity in the predictions, raising concerns about their safety.^77,79^

To understand the stability of BPA analog–LXRβ complexes, MD simulations were performed and compared with the reference BPA-LXRβ and G58-LXRβ complexes. This approach helps in assessing the behavior of the receptor during ligand interaction.^80,81^ Protein RMSD analysis showed that all complexes were stabilized within 100 ns, indicating relatively stable interactions between the BPA analogs and LXRβ. Additional parameters such as ligand RMSD, RMSF, Rg, SASA, hydrogen bonds, PCA, and FEL were also analyzed to gain a deeper understanding of the complex dynamics. Comparison of the apo-LXRβ and ligand-bound simulations indicates that BPA analogs are associated with changes in the conformational dynamics of LXRβ, which could potentially affect its biological activity.^23^ While our MD simulations confirm pose stability, the 100 ns time scale may not capture all conformational events. Enhanced sampling methods or longer simulations would be required for a complete dynamic and thermodynamic profile.^82^ This represents a direction for future, more detailed investigation.

The binding affinity of BPA analogs for LXRβ was further assessed using binding free energy calculations via the MM-PBSA method.^83^ This approach estimates the binding free energy of protein–ligand complexes based on MD simulation trajectories. A higher binding affinity corresponds to more negative binding energy values, indicating stronger and more stable interactions.^84^ According to our analysis, most BPA analogs demonstrated stronger binding to LXRβ compared to BPA itself, except for 2,2′-Diallyl-4,4′-sulfonyldiphenol. The computational evidence presented here provides actionable intelligence for health risk assessment. Specifically, the higher predicted binding affinity of BPPH and BPP for LXRβ compared to the established lr ligand G58 suggests a stronger potential to disrupt LXRβ-mediated signaling. This highlights the need to prioritize these analogs for further experimental validation of their endocrine-disrupting activity.^70^ Nonalcoholic fatty liver disease (NAFLD) now affects 25% of adults globally, and BPA exposure is correlated with its severity.^74,85^ Our findings that BPA analogs may be even more potent ligands for LXRβ suggest that current chemical substitutions could inadvertently exacerbate this epidemic. However, it is important to note that binding affinity does not equate to toxicity, as bioavailability, metabolism, and receptor activation dynamics critically modulate biological outcomes.^86,87^ Although endocrine-disrupting effects of some BPA analogs are well-documented, experimental evidence specifically implicating LXRβ-mediated toxicity remains limited.^24,68^ This study helps prioritize high-risk BPA analogs for further investigation, thereby guiding experimental toxicologists toward compounds with elevated concern.^86,88^ The findings contribute to early hazard identification and could inform chemical prioritization frameworks for emerging contaminants.^86^

The timing of this research is particularly critical, given the European Union’s January 2025 ban on BPA in food contact materials,^89–91^ which will drive increased reliance on the very analogs our study identifies as potentially more hazardous. Our computational framework offers regulatory agencies a proactive tool to evaluate substitute chemicals before widespread market adoption, addressing the “regrettable substitution” phenomenon that has plagued chemical policy for decades.^92,93^ Computational screening reveals that many BPA alternatives may pose greater hepatotoxic risks than the banned parent compound, highlighting the urgent need for systematic hazard assessment of chemical substitutes before market introduction. Overall, this work advances structure–activity relationship models for nuclear receptors and supports the role of computational toxicology in risk assessment paradigms.^24,94^

The convergence of computational predictions with emerging regulatory actions underscores the timeliness of this research. As the pharmaceutical industry increasingly relies on molecular docking for drug discovery,^95–97^ applying these same rigorous computational approaches to environmental health hazards represents a paradigm shift toward predictive toxicology.^98,99^ Our framework offers a proactive alternative to reactive chemical regulation by using molecular docking and dynamics to predict how structural analogs bind to a target like LXRβ. This predicted binding event represents a potential molecular initiating event for endocrine disruption, which can be flagged before downstream toxic effects are observed *in vivo*.

## 5 Conclusion

Liver X Receptors (LXRs), a member of the nuclear receptor superfamily, play a key role in regulating cholesterol, glucose, and lipid metabolism, as well as inflammation. Previous studies have shown that BPA can interfere with the normal function of LXRs. In this study, a range of computational approaches was employed to investigate how BPA analogs interact with LXRβ in comparison to BPA and the known LXRβ ligand G58. Our findings suggest that several BPA analogs exhibit stronger predicted binding affinity toward LXRβ than BPA. In addition, their pharmacokinetic and pharmacodynamic properties were evaluated, and key amino acid residues involved in the interactions were identified. This study provides new insights into the structure–activity relationships between BPA analogs and LXRβ. To our knowledge, this is the first report to explore these interactions in detail across multiple BPA analogs with LXRβ. This computational study reveals that many “BPA-free” chemical alternatives may bind more strongly to a key liver metabolic receptor than BPA itself, suggesting these substitutes may pose equal or greater hepatotoxic risks. The research provides regulatory agencies with a rapid screening framework to identify potentially hazardous chemical substitutes before widespread market adoption, addressing a critical gap in chemical safety assessment that could prevent the next wave of endocrine-disrupting exposures.

## Supplementary Material



## Notes

Figure S1: An AOPWIKI-EXPLORER network visualization linking BPA and its structural analogs to adverse outcome pathways. Figures S2–S4: Detailed molecular dynamics analyses, including the Solvent Accessible Surface Area (SASA) profile (Figure S2) and Free Energy Landscape (FEL) plots for LXRβ in its apo state and in complex with all ligands (Figures S3 and S4). Table S1: Comprehensive summary of simulation results (RMSD, RMSF, Rg, SASA) for the comparative analysis of structural stability and flexibility (PDF)

## Notes

R.K.P.: Conceptualization, Methodology, Software, Formal analysis, Investigation, Writing – original draft, Writing – review and editing. S.K.: Validation, Writing – review and editing. V.K.: Conceptualization, Investigation, Resources, Supervision, Writing – review and editing, Project administration, Funding acquisition
