# Supplementary material for: A Computational Framework to Evaluate Interactions of BPA and Its Analogs with Human Liver X Receptor-Beta for Health Risk Assessment
Source: Chem Res Toxicol. 2026 Feb 16;39(3):361–75. doi: 10.1021/acs.chemrestox.5c00460 (PMC12997255; doi:10.1021/acs.chemrestox.5c00460)
Supplement: Supplementary file 1 [file tx5c00460_si_001.pdf]

Supporting Information

Computational framework to evaluate interactions of BPA and its analogs with human liver X receptor-beta for health risk assessment

Rajesh Kumar Pathak[1,2], Saurav Kumar[1,3] and Vikas Kumar[1,2,3*]

[1]Pere Virgili Institute for Health Research, Tarragona, 43005, Spain

[2]Department of Chemical Engineering, Rovira i Virgili University, Tarragona, 43007, Spain

[3]German Federal Institute for Risk Assessment, Berlin, 10589, Germany

***Corresponding author:** vikas.kumar@urv.cat

Supplementary Figures

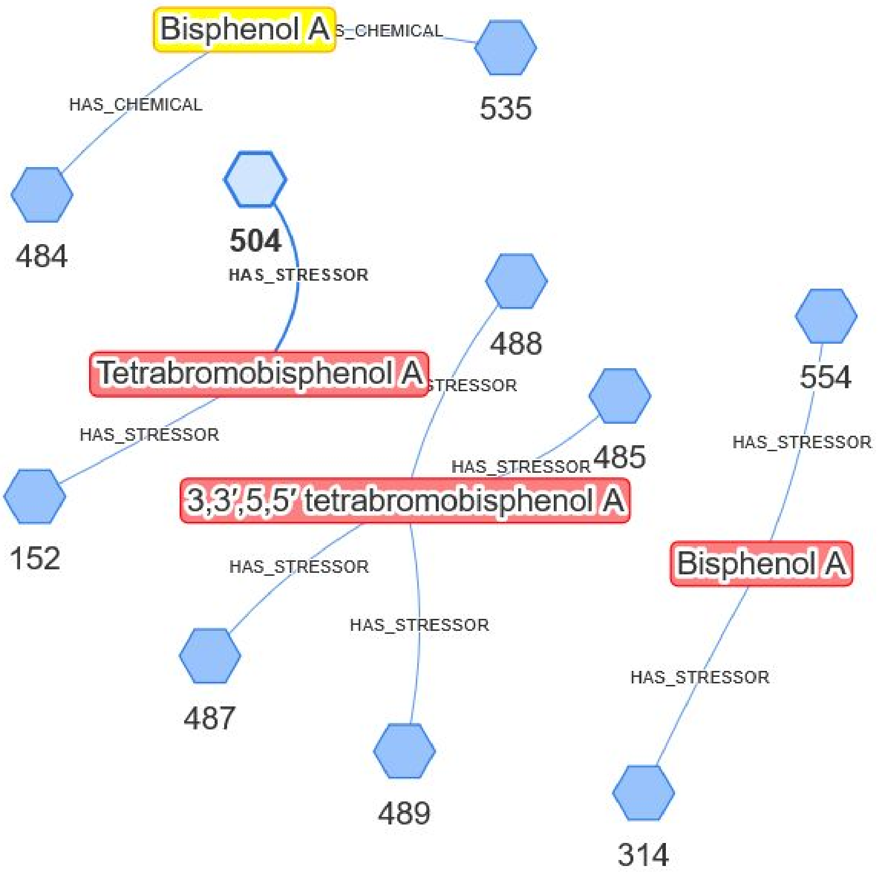

Figure S1. AOPWIKI-EXPLORER-based visualization of AOPs linked to BPA and its structural analogs. Hexagons denote AOP IDs, and connecting lines indicate pathways in which each bisphenol acts as a stressor.

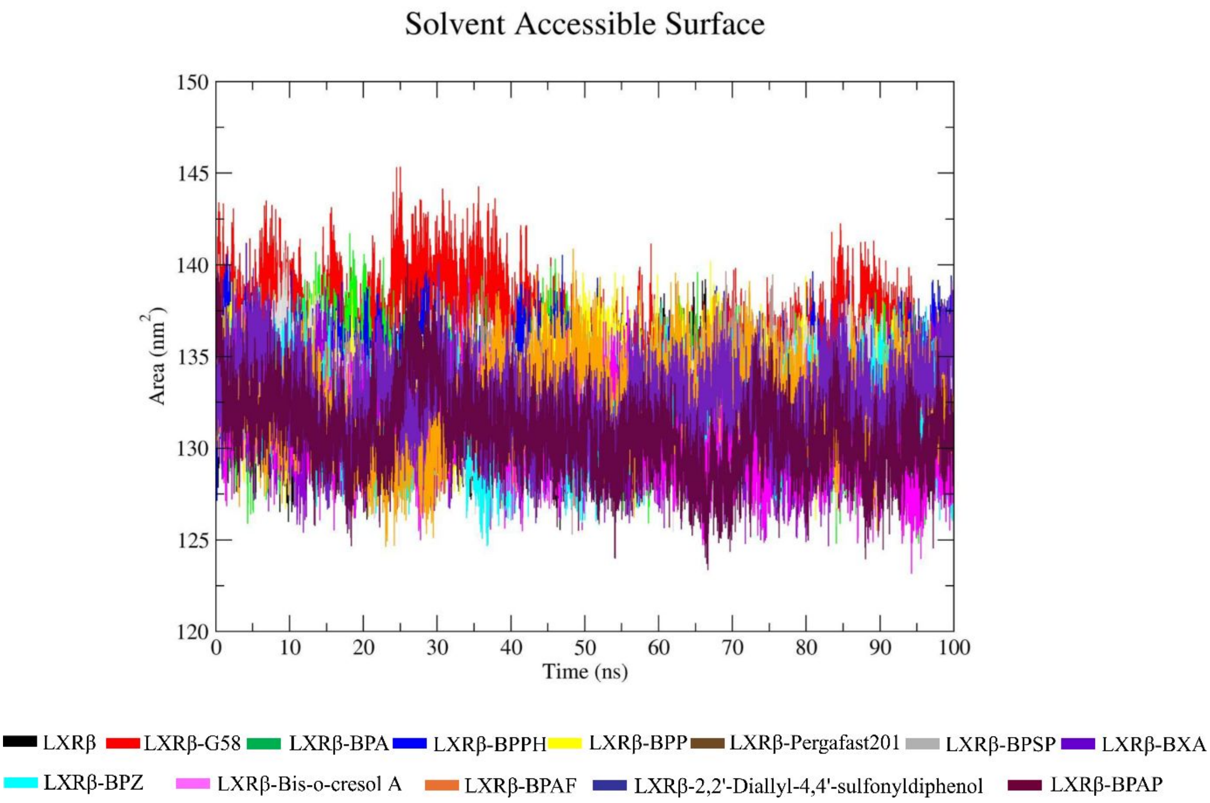

Figure S2. Solvent Accessible Surface Area (SASA) analysis during molecular dynamics simulation. SASA analysis was performed over the 100 ns simulation timeframe to evaluate the effect of ligand binding on the solvent exposure of LXR. Variations in SASA reflect changes in the compactness and conformational flexibility of LXR upon interaction with G58, BPA, and its analogs.

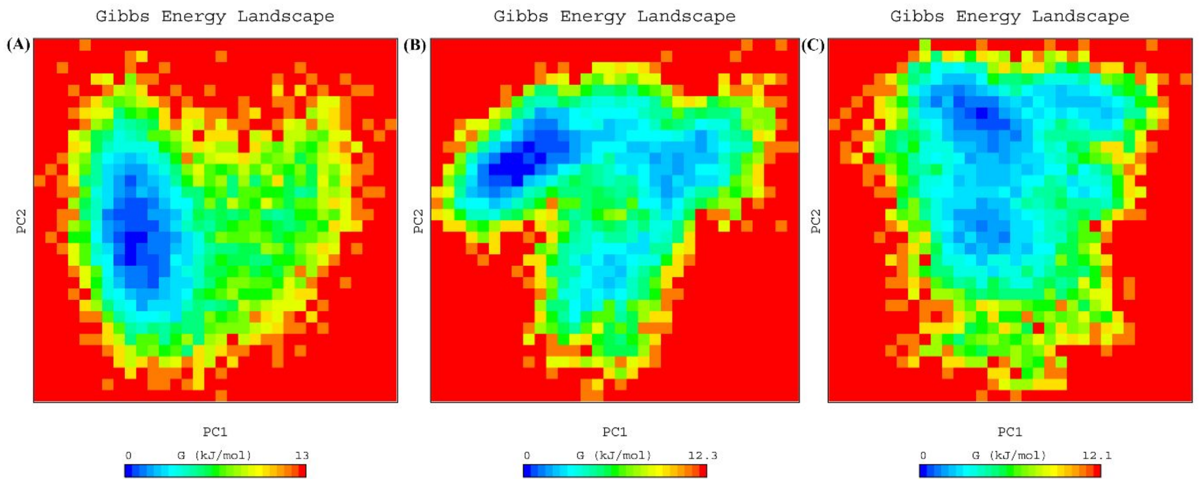

Figure S3. Free energy landscape (FEL) analysis of LXR and its ligand-bound complexes. The plots are color-coded based on Gibbs free energy (kJ mol^{-1}), mapped along the first two principal components (PC1 and PC2), representing major conformational motions. Blue regions indicate the most stable (lowest energy) states, while red regions indicate higher energy conformations. (A) LXR, (B) LXR–G58 complex, (C) LXR–BPA complex.

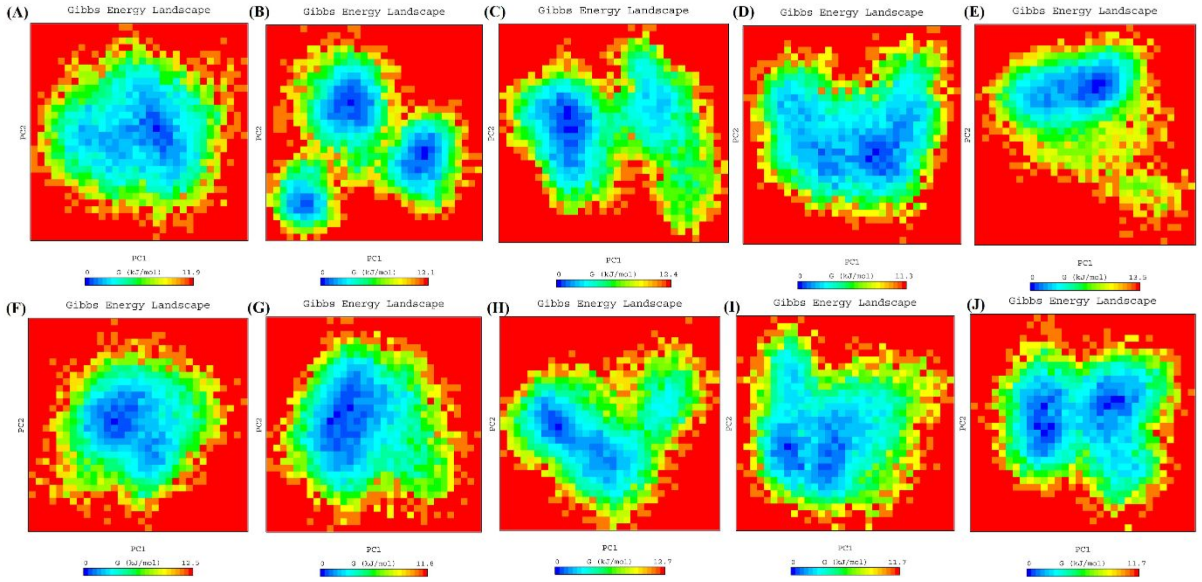

Figure S4. Free energy landscape (FEL) analysis of LXR and its ligand-bound complexes. The plots are color-coded based on Gibbs free energy (kJ mol^{-1}), mapped along the first two principal components (PC1 and PC2), representing major conformational motions. Blue regions indicate the most stable (lowest energy) states, while red regions indicate higher energy conformations. (A) BPPH, (B) BPP, (C) Pergafast201, (D) BPSP, (E) BXA, (F) BPZ, (G) Bis-o-cresol A, (H) BPAF, (I) 2,2′-Diallyl-4,4′-sulfonyldiphenol and (J) BPAP

Table S1. Comparative analysis of structural stability and flexibility for LXRβ and all its complexes. The table summarizes key MD parameters: protein backbone RMSD, ligand RMSD, RMSF, radius of gyration (Rg), and SASA values.

System	Protein RMSD (nm)	Ligand RMSD (nm)	RMSF	Rg	SASA
LXRβ	0.11		0.07	1.83	132.07
LXRβ-G58	0.16	0.05	0.08	1.85	136.07
LXRβ-BPA	0.14	0.10	0.08	1.84	132.92
LXRβ-BPPH	0.16	0.16	0.08	1.85	133.65
LXRβ-BPP	0.12	0.12	0.08	1.84	132.94
LXRβ-Pergafst201	0.11	0.20	0.08	1.84	132.48
LXRβ-BPSP	0.11	0.18	0.08	1.84	132.48
LXRβ-BXA	0.11	0.21	0.07	1.83	131.13
LXRβ-BPZ	0.12	0.07	0.07	1.83	131.61
LXRβ-Bis-o-cresol A	0.11	0.12	0.07	1.84	130.94
LXRβ-BPAF	0.12	0.09	0.08	1.84	132.56
LXRβ- 2,2'-Diallyl-4,4'-sulfonyldiphenol	0.11	0.12	0.08	1.83	132.54
LXRβ-BPAP	0.11	0.06	0.07	1.83	130.83
